# Extracapsular dissection versus superficial parotidectomy: real-world comparison of postoperative complications and facial nerve injury

**DOI:** 10.1097/JS9.0000000000005383

**Published:** 2026-05-01

**Authors:** Yi-Chan Lee, Cheng-Ming Luo, Li-Jen Hsin, Yao-Te Tsai, Rodney Cheng-En Hsieh, Tsung-You Tsai, Shiow-Ing Wang

**Affiliations:** a Department of Otolaryngology - Head and Neck Surgery, Chang Gung Memorial Hospital, Keelung, Taiwan; b College of Medicine, Chang Gung University, Taoyuan, Taiwan; c Department of Otolaryngology - Head and Neck Surgery, Chang Gung Memorial Hospital, Taoyuan, Taiwan; d Department of Otolaryngology - Head and Neck Surgery, Chang Gung Memorial Hospital, Chiayi, Taiwan; e Department of Radiation Oncology, Chang Gung Memorial Hospital, Taoyuan, Taiwan; f Center for Health Data Science, Department of Medical Research, Chung Shan Medical University Hospital, Taichung, Taiwan; g Department of Health Policy and Management, College of Health Care and Management, Chung Shan Medical University, Taichung, Taiwan

**Keywords:** extracapsular dissection, facial nerve, parotidectomy

## Abstract

**Background:**

Superficial parotidectomy (SP), involving routine facial nerve (FN) dissection, is the standard approach for benign superficial parotid tumors. Extracapsular dissection (ECD) offers a less invasive alternative by avoiding formal FN identification, but large-scale comparative data remain limited.

**Objective:**

To compare postoperative complication rates between ECD and SP.

**Methods:**

This retrospective study used the TriNetX database to identify adults with benign superficial parotid tumors who underwent ECD or SP. Propensity score matching (PSM) was performed to balance baseline covariates. Primary outcomes within 30 days included overall FN injury, surgical site infection, wound disruption, postoperative bleeding, and salivary fistula. Secondary outcomes were all-cause mortality, hospital admission, and emergency department visits within 30 days. Facial reanimation procedures were assessed at 180 days, and at 1, 3, and 5 years.

**Results:**

PSM yielded 1839 patients per group. ECD was associated with a significantly lower 30-day FN injury risk than SP (HR: 0.458; 95% CI: 0.273–0.768). No significant differences were observed for surgical site infection (HR: 0.674; 95% CI: 0.325–1.399), wound disruption (HR: 1.521; 95% CI: 0.541–4.273), postoperative bleeding (HR: 0.905; 95% CI: 0.471–1.742), salivary fistula (HR: 0.338; 95% CI: 0.068–1.676), all-cause mortality (HR: 1.014; 95% CI: 0.063–16.21), hospital admission (HR: 0.931; 95% CI: 0.684–1.267), or emergency department visits (HR: 1.259; 95% CI: 0.888–1.784). Facial reanimation rates were comparable at all time points.

**Conclusions:**

In this large, real-world cohort, ECD significantly reduced the early risk of FN injury compared with SP, without increasing other short-term complications or long-term facial reanimation rates. These findings support ECD as a favorable option for selected patients with benign superficial parotid tumors following shared decision-making.

## Introduction

Benign tumors account for the majority of parotid gland neoplasms, with pleomorphic adenoma and Warthin’s tumor being the most common histologic subtypes^[^1–4^]^. Most of these benign lesions are located in the superficial lobe of the gland, lateral to the facial nerve (FN)^[^3–5^]^. Surgical resection remains as the primary treatment^[^6–10^]^. Traditionally, superficial parotidectomy (SP) has been the standard approach, involving tumor removal along with the superficial lobe through routine FN dissection^[^2–5,11–15^]^. In contrast, extracapsular dissection (ECD) is a less invasive technique that excises the tumor with a small margin of normal tissue while avoiding formal FN identification^[^2–5,11–16^]^. Despite published retrospective studies and meta-analyses comparing these procedures, well-conducted randomized controlled trials are notably absent, reflecting the inherent challenges of conducting such studies^[^1–5,11–20^]^.

Population-level databases combined with propensity score matching (PSM) offer a means to simulate group randomization by balancing confounding factors^[^21–23^]^. TriNetX, an international network, provides access to large multi-institutional clinical datasets suitable for such analyses.^[^24–26^]^ This study aims to utilize TriNetX and PSM to compare postoperative complications between ECD and SP, thereby providing real-world evidence to guide surgical management of benign and superficial parotid tumors. This study adheres to the Transparency In The reporting of Artificial Intelligence (TITAN) guideline, with transparent reporting of AI-assisted tools used during manuscript preparation^[^27^]^.

## Methods

### Study design and data source

This retrospective cohort study utilized real-world clinical data from TriNetX, a global health research network compiling de-identified electronic medical records from over 250 million individuals, contributed by more than 120 healthcare organizations (HCOs) worldwide. The platform employs a standardized quality assurance framework encompassing conformance, completeness, and plausibility metrics^[^28^]^. For this analysis, data were accessed in April 2025 from the U.S. Collaborative Network, a TriNetX subnetwork composed of 68 HCOs. The dataset covered patient encounters from January 1, 2016, up to December 31, 2024.

### Ethical considerations

TriNetX complies with both the Health Insurance Portability and Accountability Act (HIPAA) and the General Data Protection Regulation (GDPR). Given its use of only aggregated and de-identified information, the platform received a waiver of informed consent from the Western Institutional Review Board (WIRB). This study was also approved by the Institutional Review Board of Chung Shan Medical University Hospital (CS2-25005).

### Patient selection

Eligible participants were adults diagnosed with benign parotid tumors, identified using International Classification of Diseases, Tenth Revision, Clinical Modification (ICD-10-CM) code D11.0, during the study period. To capture only incident cases, individuals with such diagnoses prior to December 31, 2015, were excluded. Patients were divided into two cohorts based on surgical intervention. The ECD group comprised individuals undergoing tumor excision or lateral lobe resection of the parotid gland without FN dissection, as coded by Current Procedural Terminology (CPT) 42410. The index date was defined as the date of the first ECD procedure. Patients who had experienced surgical complications (as defined below) or had died on or before the index date were excluded. The SP group comprised individuals undergoing parotidectomy of the lateral lobe of the parotid gland, involving FN dissection and preservation, as identified by CPT 42 415. The same index date definition and exclusion criteria were applied to the SP group.

### Outcome definitions

Primary outcomes comprised surgery-related complications, identified via ICD-10-CM or CPT codes, and included overall FN injury (G51, S04.5, R29.810), surgical site infection (T81.4), wound disruption (T81.3), postoperative hemorrhage (L76.2, L76.3), and salivary fistula (K11.4). Secondary outcomes included all-cause mortality, hospital admissions, and emergency department visits. Both primary and secondary outcomes were assessed from day 1 to day 30 post-surgery, as these complications typically arise during the early postoperative period. In addition, we analyzed the incidence of facial reanimation procedures, including re-innervation, identified by CPT codes 64716, 64864, 64868, 64885, 64886, 64910, 64727, 15840, 15841, 15845, 67917, 67912, and 67900^[^29,30^]^. The TriNetX platform enables calculation of procedure events over specified follow-up intervals. Because permanent FN injury may be coded even in the immediate postoperative period, making it difficult to distinguish from transient injuries, we therefore used facial reanimation procedures as a long-term indicator of FN function. These procedures were evaluated from the index day to 180 days, 1 year, 3 years, and 5 years postoperatively.

HIGHLIGHTS

- This study provides the first real-world, population-level database comparison of outcomes between extracapsular dissection (ECD) and superficial parotidectomy (SP).
- ECD was associated with a significantly reduced risk of overall facial nerve injury in the early postoperative period compared with SP. This benefit was consistent among female and non-smoking patients, with non-smokers undergoing ECD also experiencing a lower risk of surgical site infection.
- The risk of facial reanimation procedures was comparable between ECD and SP across all assessed time points.

### Covariates

To minimize confounding, covariates were assessed during the 1-year baseline period prior to surgery. We included variables across demographics, lifestyle factors, functional status, healthcare utilization, comorbidities, medication history, and laboratory data. Demographics included age, sex, race, and proxies for socioeconomic status (ICD-10-CM codes Z55-Z65). Lifestyle factors included tobacco use (Z72.0), nicotine dependence (F17), and alcohol-related disorders (F10). Functional status was assessed via mobility-related ICD-10-CM codes (Z99.3, Z74.0). Healthcare utilization variables included prior outpatient care (CPT 1013626), emergency services (1013711), inpatient or observation care (1013659), and preventive services (1013829). Comorbidities were captured as binary variables using ICD-10-CM codes, including hypertension (I10–I1A), ischemic heart disease (I20–I25), heart failure (I50), cerebrovascular disease (I60–I69), vascular disorders (I70–I79), diabetes (E08–E13), malnutrition (E40–E46), vitamin D deficiency (E55), obesity (E66), hyperlipidemia (E78.5), chronic kidney disease (N18), liver disease (K70–K77), hematologic and immune disorders (D50–D89), and viral hepatitis (B15–B19). Medication history comprised statin use (ATC code C10AA), non-steroidal anti-inflammatory drugs (M01A), aspirin (N02BA01), and systemic corticosteroids (H02). Laboratory data, including body mass index, leukocyte count, and differential white blood cell percentages (neutrophils, lymphocytes, monocytes, eosinophils, and basophils) were also captured.

### Statistical analysis

To reduce confounding bias, PSM was performed using TriNetX’s integrated tools. A 1:1 nearest-neighbor matching algorithm, employing a caliper of 0.1 pooled standard deviations, was utilized to balance baseline characteristics. Group comparability before and after matching was assessed using propensity score density plots and standardized mean differences (SMDs), with values <0.1 considered indicative of adequate balance^[^31^]^. Kaplan–Meier survival curves were generated to estimate the cumulative incidence for each outcome. Hazard ratios (HRs) and 95% confidence intervals (CIs) were calculated using the Survival package in R (v3.2-3). The proportional hazards assumption was tested using the generalized Schoenfeld residual method, as implemented within TriNetX. Differences between survival curves were assessed via the log-rank test. Subgroup analyses were performed by sex (male versus female), age group (18–64 years versus ≥65 years), smoking, obesity, and diabetes mellitus. Sensitivity analyses were also conducted by adjusting the matching covariates. For all analyses, no imputations were performed for missing data. Continuous and categorical variables were reported using the number of available observations and the corresponding percentages.

## Results

### Characteristics of study participants

**Figure 1.**
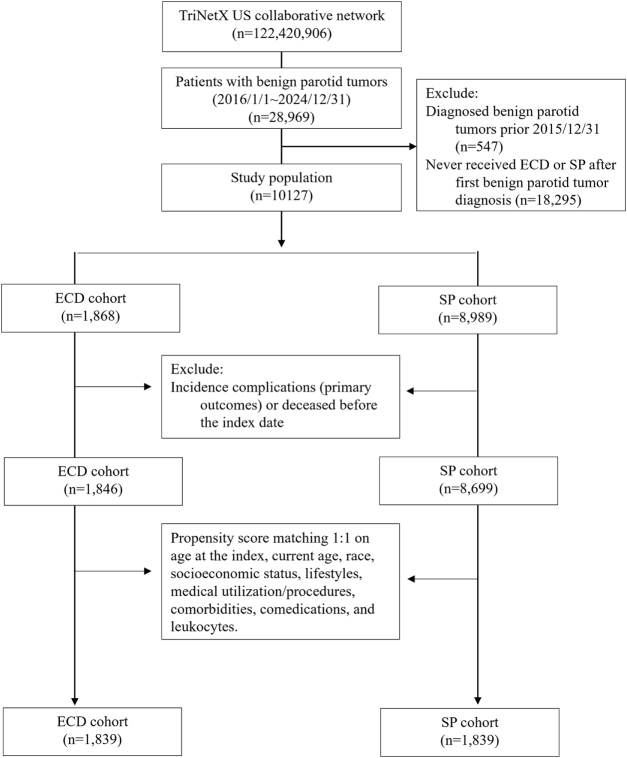
Study flowchart.

A total of 1846 individuals who underwent ECD and 8699 who received SP were identified for inclusion. After performing 1:1 PSM, 1839 patients remained in each group. The cohort selection process is outlined in Figure 1. Table 1 presents the baseline characteristics before and after matching. Prior to matching, the two groups were generally comparable. Following matching, all covariates achieved satisfactory balance, with SMDs below 0.1. The propensity score density plots demonstrated substantial overlap between the ECD and SP cohorts after matching, confirming adequate baseline balance (Supplemental Digital Content eFigure 1, available at: http://links.lww.com/JS9/H397). Median follow-up duration was consistent across cohorts, namely 365 days for both groups, with interquartile ranges of 231 days for ECD and 229 days for SP.

**Table 1:** Baseline characteristics of study subjects (before and after PSM matching).

Variables	Before PSM a			After PSM a		
	ECO cohort (*n* = 1846)	SP cohort (*n* = 8699)	SMD	ECO cohort (*n* = 1839)	SP cohort (n = 1839)	SMD
Current age, mean ± SD	61.7 ± 15.6	61.1 ± 15.7	0.038	61.7 ± 15.6	62.0 ± 15.0	0.017
Age at index, mean ± SD	57.1 ± 15.5	56.8 ± 15.6	0.021	57.1 ± 15.6	57.4 ± 15.0	0.021
Sex, *n* (%)						
Female	1024 (55.5)	4593 (52.8)	0.054	1022 (55.6)	1012 (55.0)	0.011
Male	781 (42.3)	3787 (43.5)	0.025	777 (42.3)	786 (42.7)	0.010
Unknown gender	41 (2.2)	319 (3.7)	0.086	40 (2.2)	41 (2.2)	0.004
Race, *n* (%)						
White	1310 (71.0)	6035 (69.4)	0.035	1308 (71.1)	1339 (72.8)	0.038
Black or African American	179 (9.7)	791 (9.1)	0.021	179 (9.7)	183 (10.0)	0.007
Unknown race	139 (7.5)	859 (9.9)	0.083	138 (7.5)	114 (6.2)	0.052
Asian	116 (6.3)	576 (6.6)	0.014	116 (6.3)	105 (5.7)	0.025
Other race	78 (4.2)	339 (3.9)	0.017	78 (4.2)	77 (4.2)	0.003
American Indian or Alaska native	14 (0.8)	32 (0.4)	0.052	10 (0.5)	12 (0.7)	0.014
Native Hawaiian or Other Pacific Islander	10 (0.5)	67 (0.8)	0.028	10 (0.5)	10 (0.5)	0.000
Social economic status, *n* (%)						
Persons with potential health hazards related to socioeconomic and psychosocial circumstances	12 (0.7)	66 (0.8)	0.013	12 (0.7)	11 (0.6)	0.007
Lifestyles, *n* (%)						
Nicotine dependence	335 (18.1)	1336 (15.4)	0.075	331 (18.0)	334 (18.2)	0.004
Tobacco use	103 (5.6)	432 (5.0)	0.027	103 (5.6)	94 (5.1)	0.022
Alcohol-related disorders	28 (1.5)	114 (1.3)	0.017	28 (1.5)	25 (1.4)	0.014
Reduced mobility	10 (0.5)	28 (0.3)	0.034	10 (0.5)	10 (0.5)	0.000
Dependence on wheelchair	10 (0.5)	10 (0.1)	0.075	10 (0.5)	10 (0.5)	0.000
Medical utilization, *n* (%)						
Office or Other Outpatient Services	1272 (68.9)	5943 (68.3)	0.013	1266 (68.8)	1270 (69.1)	0.005
Emergency Department Services	210 (11.4)	966 (11.1)	0.009	209 (11.4)	198 (10.8)	0.019
Preventive Medicine Services	163 (8.8)	824 (9.5)	0.022	163 (8.9)	155 (8.4)	0.015
Hospital Inpatient and Observation Care Services	64 (3.5)	341 (3.9)	0.024	64 (3.5)	60 (3.3)	0.012
Comorbidities, *n* (%)						
Hypertensive diseases	618 (33.5)	2781 (32.0)	0.032	614 (33.4)	625 (34.0)	0.013
Hyperlipidemia, unspecified	307 (16.6)	1474 (16.9)	0.008	305 (16.6)	313 (17.0)	0.012
Diabetes mellitus	264 (14.3)	1183 (13.6)	0.020	260 (14.1)	279 (15.2)	0.029
Overweight and obesity	181 (9.8)	895 (10.3)	0.016	180 (9.8)	170 (9.2)	0.019
Diseases of the blood and blood-forming organs and certain disorders involving the immune mechanism	161 (8.7)	786 (9.0)	0.011	161 (8.8)	164 (8.9)	0.006
Ischemic heart diseases	159 (8.6)	726 (8.3)	0.010	156 (8.5)	162 (8.8)	0.012
Depressive episode	148 (8.0)	603 (6.9)	0.041	146 (7.9)	151 (8.2)	0.010
Diseases of arteries, arterioles and capillaries	112 (6.1)	499 (5.7)	0.014	112 (6.1)	121 (6.6)	0.020
Cerebrovascular diseases	105 (5.7)	388 (4.5)	0.056	104 (5.7)	108 (5.9)	0.009
Vitamin D deficiency	100 (5.4)	455 (5.2)	0.008	100 (5.4)	97 (5.3)	0.007
Chronic kidney disease (CKD)	73 (4.0)	344 (4.0)	0.000	73 (4.0)	77 (4.2)	0.011
Diseases of liver	65 (3.5)	286 (3.3)	0.013	64 (3.5)	65 (3.5)	0.003
Heart failure	39 (2.1)	243 (2.8)	0.044	39 (2.1)	37 (2.0)	0.008
Major depressive disorder, recurrent	37 (2.0)	138 (1.6)	0.031	36 (2.0)	30 (1.6)	0.025
Viral hepatitis	21 (1.1)	70 (0.8)	0.034	21 (1.1)	15 (0.8)	0.033
Malnutrition	10 (0.5)	35 (0.4)	0.020	10 (0.5)	10 (0.5)	0.000
Co-medication, *n* (%)						
Corticosteroids for systemic use	452 (24.5)	2052 (23.6)	0.021	446 (24.3)	446 (24.3)	0.000
HMG CoA reductase inhibitors	439 (23.8)	1940 (22.3)	0.035	436 (23.7)	428 (23.3)	0.010
NSAIDs	382 (20.7)	1747 (20.1)	0.015	379 (20.6)	396 (21.5)	0.023
Aspirin	196 (10.6)	948 (10.9)	0.009	192 (10.4)	196 (10.7)	0.007
Laboratory, *n* (%)						
Body mass index (BMI, kg/m^2^)	1449 (78.5)	6634 (76.2)	0.052	1442 (78.4)	1431 (77.8)	0.014
Mean ± SD	29.74 ± 6.74	29.81 ± 6.82	0.010	29.71 ± 6.73	30.05 ± 6.91	0.050
Leukocytes in blood (10*3/µL)	918 (49.7)	4192 (48.2)	0.030	911 (49.5)	879 (47.8)	0.034
Mean ± SD	7.519 ± 2.57	8.463 ± 61.7	0.021	7.515 ± 2.56	7.554 ± 2.68	0.014
Neutrophils in blood (10*3/µL)	719 (38.9)	3102 (35.6)	0.068	714 (38.8)	680 (36.9)	0.038
Mean ± SD	40.35 ± 375	56.33 ± 471	0.037	40.59 ± 376	61.69 ± 491	0.048
Monocytes/100 leukocytes in blood (%)	752 (40.7)	3299 (37.9)	0.057	747 (40.6)	726 (39.5)	0.023
Mean ± SD	8.047 ± 2.29	8.147 ± 2.45	0.041	8.049 ± 2.29	8.027 ± 2.39	0.009
Lymphocytes/100 leukocytes in blood (%)	744 (40.3)	3171 (36.4)	0.079	739 (40.2)	697 (37.9)	0.046
Mean ± SD	28.29 ± 9.81	28.00 ± 9.55	0.030	28.30 ± 9.79	28.00 ± 9.71	0.031
Eosinophils/100 leukocytes in blood (%)	741 (40.1)	3239 (37.2)	0.059	736 (40.0)	707 (38.4)	0.032
Mean ± SD	2.661 ± 2.12	2.658 ± 2.04	0.001	2.661 ± 2.13	2.683 ± 2.14	0.010
Basophils/100 leukocytes in blood (%)	740 (40.1)	3227 (37.1)	0.061	735 (39.9)	705 (38.3)	0.033
Mean ± SD	0.648 ± 0.38	0.675 ± 0.40	0.068	0.647 ± 0.38	0.641 ± 0.40	0.014

Note: PSM: propensity score matching, ECO: Extracapsular dissection, SP: Superficial parotidectomy, SMD: Standardized mean difference, SD: Standard deviation, HMG CoA: hydroxy-3-methylglutaryl coenzyme A, NSAIDs: Anti-inflammatory and antirheumatic products, non-steroids.

If the patient is less or equal to 10, results show the count as 10.

^a^ Propensity score matching was performed on age at the index, current age, sex, race, socioeconomic status, lifestyles, medical utilization/procedures, comorbidities, and Leukocytes.

### Primary outcomes

**Figure 2.**
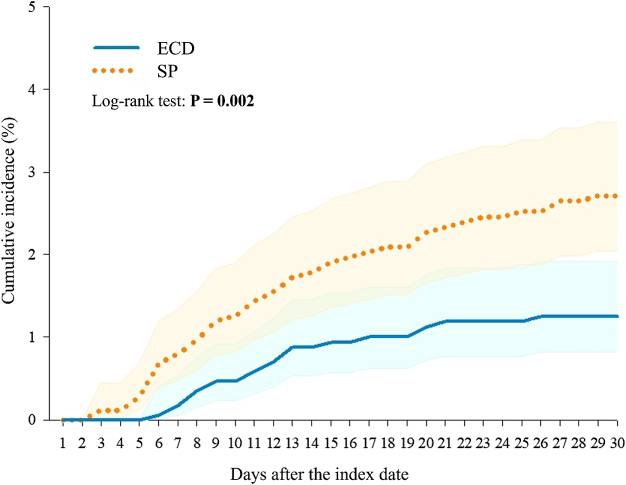
Kaplan–Meier curve for cumulative incidence of overall facial nerve injury.

Table 2 displays the incidence of primary outcomes and their associated HRs within 30 days postoperatively. The ECD group demonstrated a significantly lower risk of overall FN injury when compared with the SP group (HR: 0.458; 95% CI: 0.273–0.768). Kaplan–Meier curves illustrated a significant divergence in the cumulative incidence of overall FN injury between the two cohorts (log-rank test, *P* = 0.002; Figure 2). For other complications, no significant intergroup differences were observed, including surgical site infection (HR: 0.674; 95% CI: 0.325–1.399), wound disruption (HR: 1.521; 95% CI: 0.541–4.273), postoperative bleeding (HR: 0.905; 95% CI: 0.471–1.742), and salivary fistula (HR: 0.338; 95% CI: 0.068–1.676). The corresponding Kaplan–Meier curves are presented in Supplemental Digital Content eFigure 2–5, available at: http://links.lww.com/JS9/H397. Analyses of facial reanimation procedures at 180 days, 1 year, 3 years, and 5 years results were comparable between the two groups, with HRs of 1.000, 1.082, 0.997, and 0.995, respectively (Supplemental Digital Content Table S1, available at: http://links.lww.com/JS9/H397). The corresponding Kaplan–Meier curve is presented in Supplemental Digital Content eFigure 6, available at: http://links.lww.com/JS9/H397.

**Table 2:** Risk of outcomes (1 day to 30 days).

Outcomes	ECD cohort (*n* = 1839)		SP cohort (*n* = 1839)		Hazard ratio (95% CI) a
	Patients with outcome	Absolute event rates (%)	Patients with outcome	Absolute event rates (%)	
Primary outcomes					
Facial nerve injury	21	1.142	46	2.501	**0.458 (0.273–0.768)**
Surgical site infection	12	0.653	18	0.979	0.674 (0.325–1.399)
Wound disruption	10	0.544	10	0.544	1.521 (0.541–4.273)
Bleeding	17	0.924	19	1.033	0.905 (0.471–1.742)
Salivary fistula	10	0.544	10	0.544	0.338 (0.068–1.676)
Secondary outcomes					
All-cause mortality	10	0.544	10	0.544	1.014 (0.063–16.21)
Inpatient/Hospitalization	78	4.241	84	4.568	0.931 (0.684–1.267)
Emergency department visit	71	3.861	57	3.100	1.259 (0.888–1.784)

ECD: Extracapsular dissection, SP: Superficial parotidectomy, CI: Confidence interval.

If the patient is less or equal to 10, results show the count as 10.

^a^ Propensity score matching was performed on age at the index, current age, sex, race, socioeconomic status, lifestyles, medical utilization/procedures, comorbidities, and leukocytes.

### Secondary outcomes

No statistically significant differences were observed between the cohorts in terms of all-cause mortality, inpatient admissions, or emergency department visits (HRs: 1.014, 0.931, and 1.259, respectively; see Table 2).

### Subgroup analyses

**Figure 3.**
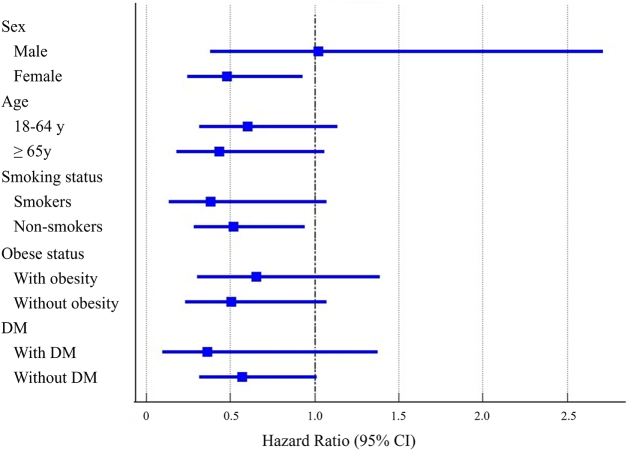
Subgroup analysis of overall facial nerve injury.

Subgroup analyses (Fig. 3, Supplemental Digital Content Table S2 and S4, available at: http://links.lww.com/JS9/H397) revealed that ECD was associated with significantly reduced risks in specific patient cohorts. Female patients in the ECD group had a significantly lower risk of overall FN injury compared to those in the SP group (HR: 0.480; 95% CI: 0.248–0.930). Similarly, among non-smokers, ECD significantly reduced risks of overall FN injury (HR: 0.517; 95% CI: 0.283–0.945) and surgical site infection (HR: 0.382; 95% CI: 0.169–0.862). No significant differences were observed across age groups (Fig. 3 and Supplemental Digital Content Table S3, available at: http://links.lww.com/JS9/H397), obesity status (Fig. 3 and Supplemental Digital Content Table S5, available at: http://links.lww.com/JS9/H397), or the presence or absence of diabetes mellitus (Fig. 3 and Supplemental Digital Content Table S6, available at: http://links.lww.com/JS9/H397). The -values for interaction across all subgroups were non-significant.

### Sensitivity analyses

The consistency of the results was confirmed through sensitivity analyses using different sets of matching variables. Across four models, ECD consistently showed a lower risk of overall FN injury, with HRs of 0.468, 0.419, 0.566, and 0.458, respectively (Supplemental Digital Content Table S7, available at: http://links.lww.com/JS9/H397).

## Discussion

This study demonstrated that, in the early postoperative period, ECD was associated with a significantly reduced risk of overall FN injury compared to SP. This reduction was also evident in female and non-smoking patient groups. Additionally, non-smoking patients in the ECD group had a lower risk of surgical site infection than those in the SP group. For other early postoperative complications, no significant intergroup differences were observed. The need for facial reanimation after initial surgery was also comparable between groups. To our knowledge, this is the first study to use a real-world database and PSM to compare ECD and SP.

The evolution of surgical techniques for benign and superficial parotid tumors has progressed under the influence of both technological and clinical considerations^[^2,4,18,19,32^]^. In the early era, due to lack of refined instruments and intraoperative nerve monitoring technologies, enucleation was widely adopted to minimize FN damage^[^2,4,18,19,32–34^]^. However, clinical evidence later revealed recurrence rates as high as 30% to 50% after enucleation^[^35–37^]^. This prompted the adoption of more extensive surgical procedures such as SP, involving formal FN dissection and removal of the entire superficial lobe along with the tumor to ensure oncologic completeness^[^3,4,18–20,32,33^]^. While SP effectively reduced recurrence, it also introduced new concerns such as FN injury, cosmetic deformities, and reduced saliva production^[^1,3,4,12,18–20,32,38^]^.

In the mid to late 1970s, ECD was introduced as a technique to avoid routine FN dissection while still achieving complete tumor removal, including the tumor capsule and a small rim of surrounding normal parotid tissue^[^39–41^]^. This relatively novel technique has been compared several times with the classic SP in previous studies^[^18–20^]^. However, possibly due to challenges in conducting such studies, a PubMed database search reveals no randomized controlled trials directly comparing these two surgical approaches. Nonetheless, database studies offer an opportunity to simulate randomization. By utilizing patient cohorts from TriNetX and applying PSM, we achieved substantial balance in baseline characteristics between the two surgical groups. This helps approximate some features of randomized controlled trials and reduces the impact of potential confounders, thereby improving the credibility of comparative analyses.^[^21–23^]^

Our findings demonstrated that ECD was associated with a significantly lower risk of overall FN injury compared to SP in the early postoperative period, a result that remained consistent across different analytical models. To further explore FN outcomes in specific patient groups, we conducted exploratory subgroup analyses. These analyses suggested a reduced risk among female and non-smoking patients. The FN cross-sectional area has been reported to be smaller in females, making the nerve potentially more susceptible to mechanical stress during surgery and thereby increasing the risk of functional impairment^[^42^]^. One study has also reported a higher rate of FN injury in female patients^[^43^]^. Additionally, postoperative FN dysfunction can result from impaired blood supply after surgery^[^4,44^]^. As a more conservative technique, ECD may better preserve circulation to the FN, an advantage particularly evident in non-smokers with preserved peripheral perfusion. Because smoking is a known risk factor for microvascular compromise, the circulation-related benefits of ECD, including lower infection risk, may be less pronounced in smokers^[^45–47^]^. These exploratory findings should be interpreted with caution, and further studies are needed to confirm whether meaningful differences exist between these subgroups.

Two published pairwise meta-analyses have compared the risk of permanent FN injury between ECD and SP^[^18,19^]^. One concluded that the risks were comparable^[^18^]^, while the other reported a lower rate with ECD^[^19^]^, reflecting a lack of clear consensus on this issue. Because there is no specific diagnostic code for permanent FN injury, it is difficult to determine its true incidence in the TriNetX database. As an alternative, we calculated and compared the number of patients in the ECD and SP groups who underwent facial reanimation procedures from the index day to various postoperative time points. Across all time points, the rates of reanimation showed no significant intergroup differences. In the literature on parotid malignancies, only about one-third of patients have been reported to undergo facial reanimation, and no large-scale data are currently available for benign parotid tumor surgery^[^48^]^. Our findings may help address this gap in the literature. Although our study cannot directly quantify the number of permanent FN injuries in each group, the proportion of patients undergoing reanimation may serve as a potential indicator. While imperfect, it offers limited insight into the burden of permanent FN injury following these two approaches.

In the early stages of ECD development, surgeons were still becoming familiar with the technique, and its use was largely restricted to smaller tumors in the superficial lobe^[^49^]^. With growing experience, more recent studies have shown a steady rise in the number of ECD performed annually, and some surgeons have broadened its indications to tumors of varying sizes and locations^[^50–52^]^. These trends suggest that ECD is gaining wider acceptance. However, this does not imply that the classic SP will be replaced. Instead, the two procedures should be regarded as complementary^[^53^]^. The choice of surgical technique is influenced by tumor characteristics, patient condition, clinical judgment, and surgeon experience. In the contemporary management of benign parotid tumors, a one-size-fits-all approach may no longer be adequate. An individualized strategy that tailors the surgical method to the characteristics of each case may provide better outcomes. Preoperative planning that considers anatomical variations, such as accessory parotid gland morphology, may further optimize surgical safety^[^54^]^. Based on these considerations, as well as findings from two recent studies published by our group, surgeons with appropriate experience in ECD may consider evaluating a patient’s suitability for ECD during the initial surgical planning for parotid tumors^[^55,56^]^. Given that ECD may be associated with better FN functional preservation and improved perioperative efficiency, ECD could be selected as the initial surgical approach in potential candidates. If intraoperative findings necessitate formal FN dissection, including exposure of the main trunk or branches, the procedure can be adapted accordingly. Depending on the relationship between the tumor and the FN, the initial ECD may be converted to partial SP, standard SP, or even total parotidectomy, as clinically indicated^[^53^]^.

This study has several limitations. First, tumor-specific variables such as size and anatomical location were not available in the database. These factors are important confounders in parotid surgery because they influence both the choice of surgical technique and the risk of complications. Although specimen weight was recorded, data were available for less than 1% of patients, which precluded meaningful subgroup analyses. Second, variations in data collection practices across participating institutions may have introduced selection bias or coding inaccuracies in the electronic health records. Third, procedures and outcomes were identified exclusively through CPT and ICD codes, which may result in misclassification, as operative findings were not accessible for verification. Outcomes occurring beyond 30 days may also not have been captured in our analysis. Fourth, recurrence of parotid tumors often requires long-term follow-up to detect, limiting the ability to analyze recurrence within the study period. Fifth, we were not able to directly determine the incidence of permanent FN injury because of coding limitations. Despite these limitations, this study provides valuable real-world evidence comparing surgical approaches in the management of benign superficial parotid tumors.

## Conclusion

This study demonstrated that ECD significantly reduced the risk of overall FN injury compared to SP in the early postoperative period. This reduction remained evident in female and non-smoking patients. Furthermore, non-smoking patients undergoing ECD also experienced a lower risk of surgical site infection. Rates of facial reanimation procedures were comparable between groups across multiple long-term time points. To our knowledge, this is the first investigation to compare ECD and SP using a real-world database combined with PSM, providing important evidence for the surgical management of benign superficial parotid tumors. ECD may be considered in selected cases, following shared decision-making with patients.

## Data Availability

Available upon reasonable request.
